# Primary osteosarcoma of the breast: case report

**DOI:** 10.1186/1757-1626-1-80

**Published:** 2008-08-09

**Authors:** Boutayeb Saber, Ahbeddou Nawal, Fetohi Mohamed, Errihani Hassan

**Affiliations:** 1 Department of medical oncology, National institute of oncology, Rabat, Morocco

## Abstract

Mammary sarcomas are very uncommon and make up less than 1% of all primary breast malignancies.

Primary osteosarcoma of the breast is extremely rare and represents 12.5% of mammary sarcomas. A secondary lesion from a primary osteosarcoma of the bone should be considered in the differential diagnosis. In addition, the absence of a direct connection between the tumour and the underlying skeleton is mandatory for the diagnosis.

We report a case of primary osteosarcoma of the breast occurring in young patient with fatal evolution.

## Introduction

Primary osteosarcoma of the breast is a very rare and aggressive entity, which is histologically indistinguishable from conventional osteosarcoma of the bone and other extraskeletal [1].

In this report, we describe case with this uncommon tumor.

## Case report

An 38-year-old woman with a medical history of asthma presented with a mass rising in the left breast without any pain. Physical examination at admission revealed a mobile mass in her right breast, estimated to be at least 8 cm. The mass was not attached to the sternum or the underlying rib. No axillary lymphadenopathy was detected on physical examination. The left breast was unremarkable. On mammography, the mass was well demarcated and partially calcified. An incisional biopsy was performed, the histological exam showed abundant cartilaginous proliferation varied from mature cartilage to poorly differentiated areas. Immunohistochemical studie was performed, immunoreactivity was detected for vimentine but not for for AE1/AE3, CK7, CK20, estrogen and progesterone receptors. There was also no overexpression of the HER-2/neu oncoprotein.

Liver echography, chest radiography and bone scan were normal. The patient underwent simple mastectomy, just after she presented dyspnea.

Chest radiography revealed a diffuse reticulonodular infiltration describing metastatic disease to the lung wich was confirmed by CT-scann.

The patient was treated by association of doxorubicin 50 mg per square meter and cisplatinium 100 mg per square meter every 21 day. The evaluation of the response basing on the CT scann showed a partial response after the third cycle and progression of the disease after the sixth cycle. The patient dead two months later.

## Discussion

Mammary sarcomas are very uncommon and make up less than 1% of all primary breast malignancies [1-3].

Primary osteosarcoma of the breast is extremely rare and represents 12.5% of mammary sarcomas [1].

The histogenesis of primary osteosarcoma of the breast is not clear, but an origin from totipotent mesenchymal cells of the breast stroma or a transformation from a pre-existing fibroadenoma or phyllodes tumor has been suggested [2-4]. Primary breast osteosarcomas are considered highly aggressive tumors associated with early recurrence and a propensity for haematogenous rather than lymphatic spread, most commonly to the lungs [1,2].

Mammographically, these tumors often present as a well-circumscribed dense lesion within the breast parenchyma with focal or extensive coarse calcifications[1,5]. The mammographic features may be deceptively benign [1].

Osteosarcoma of the breast, similar to other extraskeletal osteosarcomas, may have a broad primary osteosarcomas of the breast are fibroblastic, osteoblastic, osteoclastic (giant cell-rich) and fibroblastic subtypes [4-6].

The diagnosis of metaplastic mammary carcinoma should be excluded before primary breast osteosarcoma is diagnosed. The term sarcomatoid carcinoma has been adapted to reflect the appearance of a mesenchymal neoplasm in these epithelial malignancies. The term carcinosarcoma has usually been used to describe biphasic tumors composed of distinguishable malignant epithelial and sarcomatoid components with heterologous elements [4,5].

A secondary lesion from a primary osteosarcoma of the bone should be considered in the differential diagnosis. In addition, the diagnosis of primary breast osteosarcoma similar to that of other extraskeletal tumors requires the absence of a direct connection between the tumor and the underlying skeleton [7,8].

Treatment for the localized disease should include complete surgical removal of the tumor with an adequate margin. Axillary lymph node dissection is not indicated because axillary node involvement is exceptional. Whereas, for the metastatic disease the chemotherapy basing on the classic drugs (doxorubicine, ifosfamide, cisplatinium, methotrexate) in the osteosarcoma is the main treatment [8,9].

Although no standard of care for these lesions has been made because the rarity of this entity [1,7,9].

## Competing interests

The authors declare that they have no competing interests.

## Authors' contributions

BS drafted the manuscript, AN performed the case management, drafted the manuscript, FM participated in the patient's management and EH corrected the manuscript. All authors read and approved the final manuscript.

## Patient consent

The authors obtained written, informed consent from the patient for open access publication of this case report.
